# Phylodynamics of hepatitis B virus genotype B in East Asia: A population genomics analysis

**DOI:** 10.14440/jbm.2025.0084

**Published:** 2025-01-17

**Authors:** Serena Y. C. Lin, Patrick C. Y. Woo

**Affiliations:** 1 Science and Technology Policy Research and Information Center, National Applied Research Laboratories, Taipei 106, Taiwan; 2 Doctoral Program in Translational Medicine and Department of Life Sciences, National Chung Hsing University, Taichung 402, Taiwan; 3 The iEGG and Animal Biotechnology Research Center, National Chung Hsing University, Taichung 402, Taiwan

**Keywords:** HBV genotype B, Phylogeography, Most recent common ancestor, Bottleneck, East Asia

## Abstract

**Background:**

Hepatitis B virus (HBV) genotype B (HBV/B) is the predominant strain in Taiwan and several East Asian countries.

**Objective:**

The aim of this study is to use comprehensive phylogenetic analysis tools to monitor the long-term molecular evolution dynamic of HBV genotype B population in East Asia.

**Methods:**

In this study, full genome sequences of HBV with temporal information were extracted from GenBank and analyzed using the Bayesian Markov chain Monte Carlo method to identify best-fitting coalescent models.

**Results:**

Bayesian Skygrid analysis revealed a viral effective population (phylodynamic) bottleneck for HBV/B in 2003, a pattern similar to the previously described HBV genotype C (HBV/C). Despite these similarities, the viral dynamics for HBV/B and HBV/C diverged after 2005. HBV/C exhibited a marked decrease in genetic diversity across East Asia, whereas HBV/B maintained stable genetic diversity after 2005. Phylogeographic analysis using Neighbor-Joining and Bayesian maximum clade credibility trees indicated that Taiwan was likely the geographic origin of the most recent common ancestor of HBV/B in East Asia. An early clade spread to Japan and subsequently to the West Coast of the United States of America. Another clade dispersed to China, spread widely across the region, and was reintroduced to Taiwan multiple times. In contrast, HBV/C likely originated in China and spread to Japan, Korea, and Taiwan over several decades.

**Conclusion:**

This study highlights the similarities and differences between the viral dynamics and geographical evolutionary pathways between HBV/B and HBV/C.

## 1. Introduction

Hepatitis B virus (HBV) infection is highly endemic in East Asia, particularly in Taiwan, Japan, Korea, China, and Hong Kong. Chronic HBV infection is a significant risk factor for liver cirrhosis (LC) and hepatocellular carcinoma (HCC).^1^ According to data from the World Health Organization, more than 240 million chronic HBV patients are suffering from chronic HBV infection, with over 80,000 deaths annually. HBV is a partially double-stranded DNA virus with a genome size of approximately 3,200 nucleotides, although virions contain pre-genomic RNA. Based on complete genome sequences, HBV is currently subclassified into 10 genotypes, labeled A to J,^2^ with an average inter-genotype nucleotide diversity of 8%. Among these ten genotypes, chronic infection with HBV genotype C (HBV/C) has been associated with a significantly higher risk of progression to LC and HCC compared to other genotypes.^3^ In terms of prevalence, HBV genotype B (HBV/B) and HBV/C are the dominant strains circulating in East Asia. These genotypes are also the most common among Asian patients in some areas of the United States of America (USA).^4^-^6^ Recently, the first extensive molecular evolutionary study of HBV/C in East Asia was conducted.^7^

In addition to HBV/C, HBV/B is another significant HBV genotype circulating in East Asia. In Taiwan, HBV/B accounts for 68% of chronic HBV infections,^8^ whereas HBV/C is responsible for only 32% of the infections. In China, HBV/B is more prevalent in the southern provinces compared to the north. For example, it constitutes 53% and 62% of chronic HBV infections in Guangdong and Fujian provinces, respectively, while its prevalence is significantly lower in northern regions such as Beijing, Gansu, and Shandon (18%, 11%, and 0%, respectively).^9^ HBV/B can be further classified into subgenotypes with distinct geographic distribution. Subgenotypes B1 (Bj) and B2 (Ba) are common in Taiwan, Japan, China, and Vietnam. Subgenotype B3 is circulating in Indonesia, while B4 is predominant in Vietnam, and a unique subgenotype B5 has been identified in the Philippines. In addition, B6 has been reported in China and the Arctic region, while subgenotypes B7, B8, and B9 have been found on an island in Southeast Asia. A subsequent study reclassified B5, B6 (from China), B7, B8, and B9 as “quasi-subgenotype B3” of Southeast Asian and Chinese origin due to the sequence divergences mostly being <4%. Furthermore, subgenotype B6 from the Arctic was renamed as B5.

Although both HBV/B and HBV/C are highly prevalent and play significant roles in causing chronic hepatitis, LC, and HCC in Taiwan and other East Asian countries, previous experiences with other viruses indicate that different genotypes of the same virus can have diverse evolutionary histories. In light of this, we analyzed the molecular evolutionary history of HBV/B in East Asia and compared it to that of HBV/C.

## 2. Materials and methods

### 2.1. Data mining and compiling of hepatitis B viral sequences in East Asian countries

HBV genotype B sequences from East Asian countries were extensively mined from the GenBank database to compile a comprehensive dataset. The sorting criteria were based on a methodology described by Lin *et al*.^7^ to ensure compatibility with molecular clock calibration.

The preliminary data were genotyped using the Neighbor-Joining (NJ) method to construct a phylogenetic tree. The analysis employed the GTR+G nucleotide substitution model and included 1,000 bootstrap replicates, with reference strains from genotypes A to J. Calculations were conducted using MEGA6 software.^10^ The preliminary dataset for genotype B comprised 430 strains collected from China, Korea, Japan, and Taiwan, with isolation dates ranging from 1987 to 2010.

The number of HBV sequences from each East Asian country obtained from the NCBI database depended on the data submissions made by scientists and the scientific development within each country. To compile a representative dataset with epidemiological connotation and abundant phylogenetic information, the dataset was trimmed using an adjusted ratio (AR), rather than including all obtained sequences. Sequences were randomly selected based on the following criteria:

(i). Prevalence ratio in population: We considered both the overall HBV prevalence in the general population and the genotype prevalence among chronic hepatitis B patients in each country. The two factors were multiplied to generate an AR, which determined the taxon number of each country.
(ii). Highest temporal density: A greater chronological sampling time within the dataset enhances the ability to analyze coalescent processes (measuring time-to-ancestry in generations)^11^ and the most recent common ancestor (MRCA). If multiple sequences were obtained from a specific year, only two or three strains were randomly selected. If only one strain was isolated in a given year, the strain was retained.
(iii). Most abundant phylogeographic information: The geographic locations of isolates were recorded as precisely as possible. For example, if both the city and country were available in NCBI annotations, the sequence location was documented with the city’s name and its corresponding longitude and latitude (using the geographic trait format compatible with the BEAST soft package).

The AR was calculated using the proportion of genotype B in chronic HBV infections from various countries: China 17.2%,^12^ Korea 1.9%,^13^ Japan 12%,^14^ Taiwan 60%,^15^ and the USA 17%.^5^ The ratio was normalized by the prevalence of HBV in the general population of each country: China 10%,^16^ Korea 5.9%,^17^ Japan 4%,^18^,^19^ Taiwan 15%,^20^ and the USA 15%.^21^ The resulting AR for China, Korea, Japan, Taiwan, and the USA were 16: 1: 4: 90: 23. Although the USA is not an Asian country, the USA HBV strains were included in the analysis due to a background investigation indicating a high prevalence of HBV/B. Sequences from each country were randomly selected according to the AR, resulting in an East Asian countries dataset comprising 331 strains (*n* = 331).

The sequence dataset for HBV/C was obtained from the publication by Lin *et al*.^7^

Given that a reasonable data size of around 200 sequences is suggested for computational efficiency in BEAST, a final dataset consisting of 150 strains (Table 1) was trimmed from the East Asian country dataset (*n* = 331).

**Table 1:** Sequence sorting ratio and distribution of reduced dataset (*n*=150)

Country	Taiwan	Japan	China	Korea	USA
HBV prevalence in the general population (A)	15%	4%	10%	5.9%	15%
Genotype B prevalence in CHB patients (B)	68%	12%	41%	1.9%	17%
Sampling ratio (A × B)	90	4	37	1	23
Final strains (n)	72	7	36	1	34
Sampling time	1987 – 2008	1996 – 2003	2005 – 2010	2008	2005

Abbreviations: CHB: Chronic hepatitis B; HBV: Hepatitis B virus.

### 2.2. Model selection, Maximum Likelihood, Neighbor-Joining inference, and molecular clock signal test

The dataset was generated for the Maximum Likelihood (ML) tree, NJ tree, and Bayesian coalescent inference. HBV/B sequences were aligned using the MUSCLE algorithm^22^,^23^ implemented in the MEGA6 software. Genotype C sequences were used as outgroups (EU882006, EU882005, EU881996, and EU91963) to root the ML and NJ trees. Outgroups were excluded in the Bayesian phylogenetic analysis as rooting was inferred through molecular clock calibration (see below). The alignments are available on request from the authors.

The best-fitting nucleotide substitution (GTR+G) model was selected using a hierarchical likelihood ratio test^24^ with PAUP* version 4.0, developed by David L. Swofford (available from http://paup.sc.fsu.edu/downl.html). NJ and ML trees were then inferred according to the best-fitting model using MEGA6 and PhyML 3.0^25^ (http://www.atgc-montpellier.fr/phyml/), respectively. Statistical support for internal branches in the phylogeny was assessed by bootstrapping (1,000 replicates) for the NJ tree and the Shimodaira–Hasegawa approximate likelihood ratio test (SH-aLRT) for the ML tree. Phylogenies were then annotated with FigTree v1.4.0 (http://tree.bio.ed.ac.uk/software/figtree/).

### 2.3. Bayesian phylogenetic inference

The molecular clock-based Bayesian phylogenetic analysis is capable of estimating the evolutionary rate, population phylodynamics, and spread routes over time for HBV/B by incorporating sampling location traits. The analysis was conducted using the Bayesian coalescent framework implemented in BEAST v1.8.2.^26^,^27^ (http://beast.bio.ed.ac.uk/Main_Page), which uses a Markov Chain Monte Carlo (MCMC) sampling method to obtain posterior distributions of tree topologies and parameter estimates. Branching nodes with a posterior probability >0.95 are considered statistically well-supported. Six different evolutionary models were tested: strict versus relaxed molecular clock, each with a constant size or Bayesian Skygrid (non-parametric) demographic prior.^28^ Depending on the model, MCMCs were run for between 300 million to 1.5 billion generations (sampling every 0.01% of the run) until the effective sampling size of each parameter estimate (after a burn-in period of 10% to 25%, depending on the model) exceeded 200 to ensure proper mixing of the Markov chain.

### 2.4. Bayesian phylogeography

The putative location of each lineage was inferred based on the country of isolation for each sequence, alongside reconstructing the ancestral origin in the ML and NJ trees. Bayesian phylogeography, which provides a more in-depth analysis incorporating both spatial and temporal information, was also performed using BEAST with a discrete trait, symmetric substitution model employing Bayesian stochastic search variable selection. The procedures for projecting the results onto Google Earth were similar to those described in the study by Lin *et al*.^7^

To assess the molecular clock signal carried in the temporally sampled viral sequences, a cross-platform software, TempEst^29^ (formerly known as Path-O-Gen), was used to explore the association between genetic divergence over time and sampling dates. A ”non-clock” phylogenetic tree was required as input for the root-to-tip regression analysis. A negative slope in this analysis indicated little or no temporal signal in the data. In addition, root-to-tip plotting served as a quality control measure for heterochronous data.

## 3. Results

### 3.1. HBV genotype B time of the most recent common ancestor in East Asia by phylogeography

**Figure 1:**
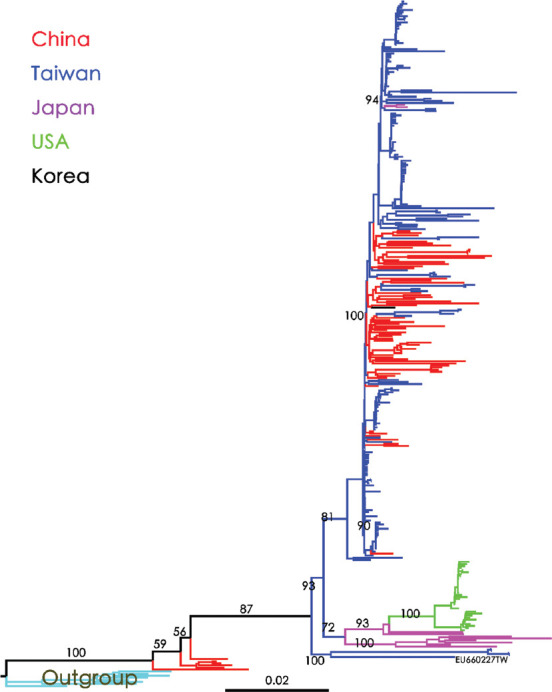
Neighbor-Joining tree reconstructed using HBV/B full genome sequences (*n* = 331) from East Asian countries. The numbers on the branches represent the bootstrap values. Outgroup sequences (genotype C) are marked light blue for outgroup rooting. Countries are specified according to color. Abbreviations: HBV/B: Hepatitis B virus genotype B.

**Figure 2:**
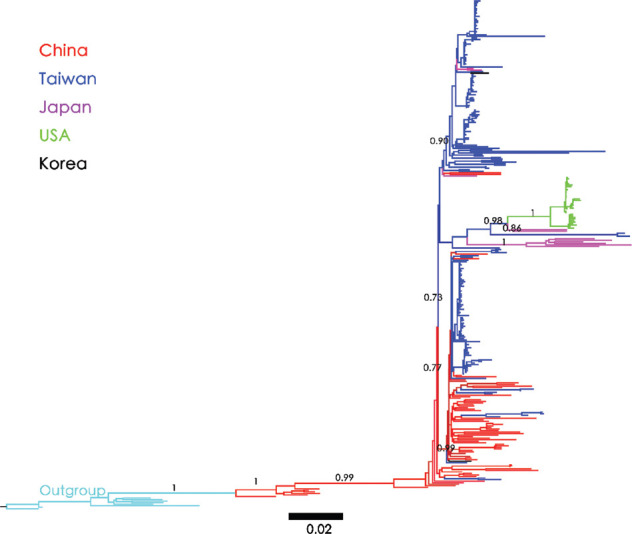
Maximum Likelihood tree reconstructed using HBV/B full genome sequences (*n* = 331) from East Asian countries. The numbers on the branches represent likelihood ratio test values (in the format of 0.XX). Outgroup sequences (genotype C) are marked light blue for outgroup-rooting. Countries are specified according to color. Abbreviations: HBV/B: Hepatitis B virus genotype B.

The NJ tree of the East Asian country dataset (*n* = 331) was rooted with HBV/C as the outgroup to infer the phylogeography of HBV/B. The analysis indicated that the origin of HBV/B in East Asia, supported by a bootstrap value of 100 (bt = 100), is Taiwan. HBV/B was introduced to China multiple times from Taiwan, and subsequently, Chinese clades migrated back and spread to Taiwan. Another branch was introduced into Japan (bt = 72) and subsequently into the USA (Figure 1). In contrast, the ML tree revealed a different pattern. In this analysis, Chinese strains appeared to be the root, spreading to Taiwan within a lower clade. (Figure 2), while the upper clade was independently constructed using Taiwanese strains (bt = 0.90). Following the conventions of phylogenetic analysis, when topologies derived from different methods do not align, the majority consensus from various analyses determines the final result.

The sequences analyzed in the Bayesian phylogeny using TempEst showed a positive regression slope for the molecular clock signal, with a correlation coefficient of 0.091, indicating that the sequences contained sufficient temporal signal. A comparison of Bayes factors across different clock models implemented in BEAST is listed in Table 2. This analysis utilized path sampling/stone sampling methods to estimate the marginal likelihood, suggesting that the “relaxed Skygrid” model is the most appropriate for this dataset.

**Table 2:** Bayes factor comparison of coalescent models for the HBV/B dataset

Dataset	N*^a^*	Model*^b^*	PS*^c^*	SS*^d^*
Reduced dataset	150	S_Const	−20357.1	−20361.0
		S_Skg	2−20323.	2−20327.
		R_Const	65−20172	11−20175
		*R_Skg	.38−2014	.45−2014
			6.62	9.52

Notes:

* Indicates the best-fitting model within this dataset. aNumber of sequences included in the data set. bBayesian Coalescent model (molecular clock model_demographic prior). cMarginal likelihood of the model estimated via path sampling method. dMarginal likelihood of the model estimated via stepping stone sampling method.

Abbreviations: Const: Constant population size; R: Relaxed clock; S: Strict clock; Skg: Non-parametric Skygrid population prior.

**Figure 3:**
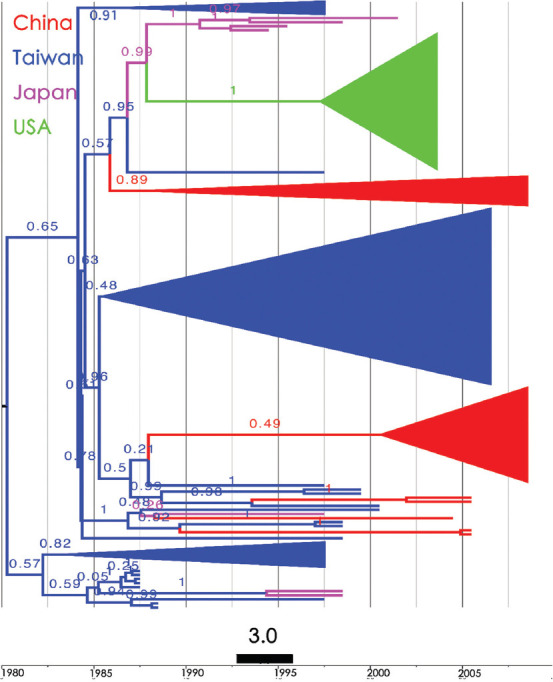
The Bayesian maximum clade credibility tree was reconstructed using HBV/B full genome sequences across East Asian countries (*n* = 150). Posterior probability (in the format of 0.XX) indicates the statistical results of branching. Countries are specified according to color. Abbreviation: HBV/B: Hepatitis B virus genotype B.

The Bayesian Maximum Clade Credibility (MCC) tree (Figure 3) constructed using the reduced dataset reveals a consistent pattern: the time of the MRCA (tMRCA) was for Taiwanese strains that diverged around 1980s and was subsequently introduced into China at several time points. One clade diverged after 1986 and spread to China, Japan, and eventually into the USA.

The similar tree topology observed in both the East Asian countries dataset and the reduced dataset indicated that a reasonable taxon (*n* < 200) with sufficient time information could yield consistent and accurate phylogenetic results.

### 3.2. Hepatitis B viral dissemination

**Figure 4:**
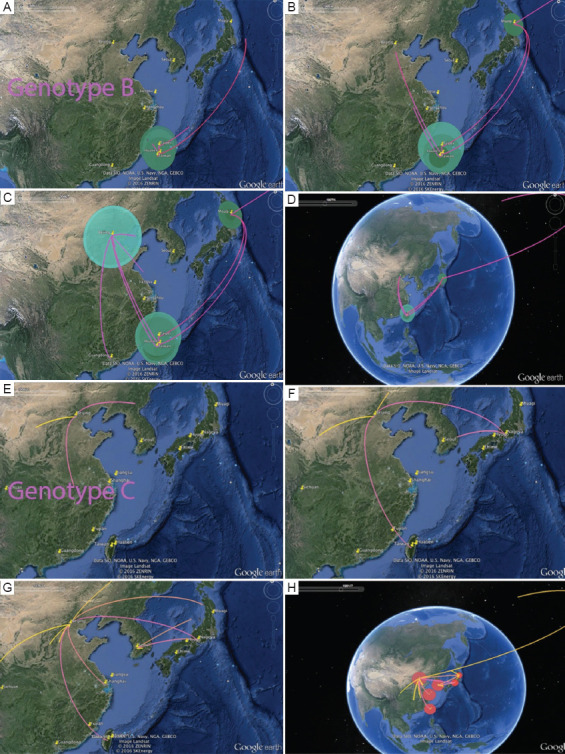
HBV/B and HBV/C phylogeography in East Asian countries visualized using Google Earth. Abbreviations: HBV/B: Hepatitis B virus genotype B; HBV/C: Hepatitis B virus genotype C.

In the Bayesian phylogeographic analysis with location trait, the animation illustrated the MRCA and transmission routes of HBV/B, supported by a Bayes factor of more than three. The tMRCA of HBV/B was located in Taiwan in the early 1980s (Figure 4A), from where it spread to China and Japan (Figure 4B). Subsequently, the virus was reintroduced from China back to Taiwan, while other strains disseminated in Japan before migrating to the USA (Figure 4C and D).

**Figure 5:**
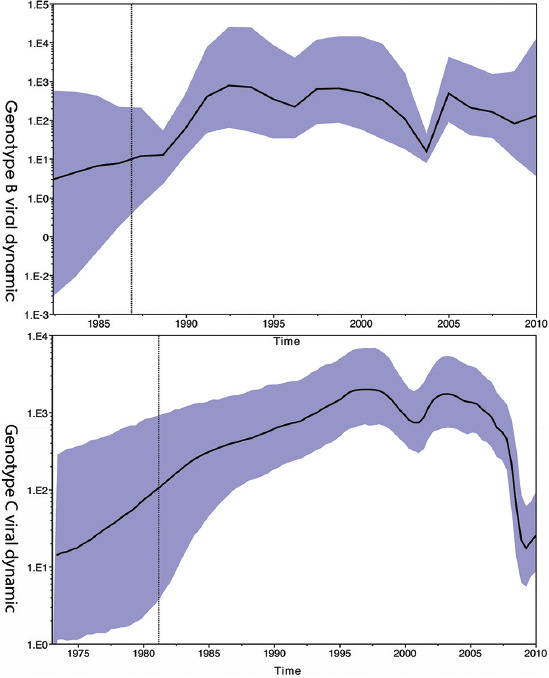
HBV/B and HBV/C genetic diversity in East Asian countries visualized using Skygrid analysis. Abbreviations: HBV/B: Hepatitis B virus genotype B; HBV/C: Hepatitis B virus genotype C.

Viral population phylodynamics illustrate the effective population size, or in other words, the genetic diversity of the population over time. In Figure 5, the genetic diversity of HBV/B increased in the late 1980s, experiencing a slight decline around 1997, and exhibited a bottleneck effect in 2003.

## 4. Discussion

In this study, we utilized publicly available HBV/B and HBV/C sequence data to demonstrate that the viral dynamics of these two HBV genotypes were similar from the 1980s until around 2005. HBV/B is the predominant genotype in Taiwan, whereas HBV/C is the predominant genotype in China.^30^ As illustrated in Figure 5, there was a notable increase in viral genetic diversity for both HBV/B and HBV/C genotypes during the 1980s. This increase appears contradictory to the commencement of the universal HBV mass vaccination program in the mid to late 1980s, which would typically be expected to reduce the HBV population and, consequently, its viral diversity. However, we propose that this apparent increase in viral genetic diversity may be attributed to an increase in gene sequencing efforts during the 1980s, leading to a greater accumulation of HBV sequence data. Following this initial phase of growth, it is noteworthy that both HBV/B and HBV/C genotypes experienced a drop in the viral population in 2003 (Figure 5). We hypothesize that this decline could be influenced by the severe acute respiratory syndrome (SARS) epidemic in 2003. The heightened fear of contracting the highly fatal SARS coronavirus resulted in increased social distancing and a significant reduction in various forms of unprotected casual sexual contact, one of the primary risk factors for human-to-human transmission of HBV. Furthermore, there was also a decrease in the frequency of long-distance travel, which contributed to a slowdown in the spread of the virus. After this transient drop in 2003, the viral genetic diversity of both HBV genotypes regained momentum and increased again over the following years as social activities and travel gradually returned to pre-epidemic levels (Figure 5).

Despite their similarities in the initial phases, the viral dynamics for HBV/B and HBV/C diverged after 2005. Following 2005, there was a marked decrease in the viral genetic diversity HBV/C in East Asian countries (Figure 5). In contrast, the viral genetic diversity of HBV/B remained stable after 2005 (Figure 5). We hypothesize that the differing trajectories of the two genotypes resulted from the interplay of two key factors. First, the universal HBV mass vaccination program has effectively suppressed the viral population and, consequently, its viral genetic diversity. Second, there has been an exponential rise in sequencing data due to advancements in sequencing technology during this period. Initially, Sanger DNA sequencing required labor-intensive processes such as labeling bases with radioactive phosphorus, running long polyacrylamide sequencing gel, developing X-ray films, and manually interpreting results. However, at the turn of the millennium, automated sequencers for Sanger sequencing were developed, allowing for automation of the manual steps. Furthermore, the robustness of the sequencing machine improved, enabling them to handle multiple samples simultaneously in the subsequent years. This remarkable improvement in Sanger sequencing technology has led to an increase in DNA sequencing data across various fields, as evidenced by the rapid expansion of the GenBank database. Therefore, we hypothesize that the relatively stable genetic diversity of HBV/B after 2005 can be attributed to the balancing effects of two factors: (i) the mass vaccination program suppressing viral genetic diversity and (ii) the improved sequencing technology increasing the apparent viral genetic diversity. In contrast, for HBV/C, the reduction in viral genetic diversity appears to be due to a stronger influence of the mass vaccination program compared to the improved sequencing technology.

In addition to their differing viral dynamic pathways after 2005, the geographical evolutionary pathways of HBV/B and HBV/C also diverged. In our previous study, we demonstrated that HBV/C originated in China using multiple algorithms^7^ subsequently spreading to Japan and Taiwan. The Japanese lineage was further disseminated to Korea, resulting in significant mixing between the Japanese and Korean lineages.^7^ In contrast, our present study indicates that the origin of HBV/B remains inconclusive when applying the same three algorithms used to analyze the origin of HBV/C.

Firstly, as shown in Figure 3, when HBV/B sequences were used to construct the Bayesian MCC phylogenetic tree, with midpoint rooting, the Taiwanese sequences (blue lines) were observed to serve as the tree root, forming two clades. The lower clade (95% highest probability density (HPD) = 0.82) formed its own phylogeny, while the upper clade (95% HPD = 0.65) spread first to China (95% HPD = 0.89), then to Japan (pink lines) and subsequently to the USA (green clade). Over time, HBV/B was introduced to China multiple times. Second, when HBV/B and HBV/C (used as outgroup) sequences were used for the construction of the NJ tree with outgroup rooting, it was shown that the outgroup sequences and some Chinese sequences (bt = 87; red lines) formed the tree root. Taiwanese sequences (bt = 100; EU660227) initiated the phylogeny of HBV/B, flowing to Japan (bt = 72; pink lines) and then to the USA (bt = 100; green lines). Subsequently, HBV/B spread to China (bt = 100; red lines) and circulated back to Taiwan several times, eventually forming another Taiwanese clade (bt = 94). Although some Chinese sequences (colored in red) clustered near the root, their bootstrap values were insufficient (below 75%) to support them as an independent clade (Figure 1).

Third, the construction of the ML tree using HBV/B and HBV/C revealed that the Chinese clade serves as the tree root (LRT = 0.99), which is the closest to the outgroup. This clade initiated the topology of HBV/B by spreading to Taiwan (LRT = 0.99), followed by a second introduction to Taiwan (LRT = 0.90), then to Japan (LRT = 1.00) and the USA (LRT = 0.98) (Figure 2). Given the conflicting results of these phylogenetic analyses, the origin of HBV/B remains elusive. Nevertheless, irrespective of whether its origin is traced back to China or Taiwan, it likely spread to Japan in the 1980s and 1990s (Figure 4) before subsequently spreading to the West Coast of the USA, probably due to migration from China (Figure 4).

## 5. Conclusion

Based on the results of the present study, it is demonstrated that the viral dynamics for HBV/B and HBV/C were similar before 2003. After the bottleneck in 2003, however, they diverged significantly: HBV/B maintained a steady state in East Asia, while HBV/C encountered a bigger bottleneck in 2009.

## Data Availability

Data used in this work are available from the corresponding author on reasonable request.
